# Serum levels of high mobility group box‐1 protein (HMGB1) and soluble receptors of advanced glycation end‐products (RAGE) in depressed patients treated with electroconvulsive therapy

**DOI:** 10.1002/npr2.12358

**Published:** 2023-06-19

**Authors:** Hiromi Abe, Mami Okada‐Tsuchioka, Naoto Kajitani, Wataru Omori, Kei Itagaki, Chiyo Shibasaki, Shuken Boku, Tetsuaki Matsuhisa, Minoru Takebayashi

**Affiliations:** ^1^ Division of Psychiatry and Neuroscience Institute for Clinical Research, National Hospital Organization (NHO) Kure Medical Center and Chugoku Cancer Center Kure, Hiroshima Japan; ^2^ Department of Pharmacy National Hospital Organization (NHO) Kure Medical Center and Chugoku Cancer Center Kure, Hiroshima Japan; ^3^ Department of Neuropsychiatry, Faculty of Life Sciences Kumamoto University Kumamoto Japan

**Keywords:** depression, electroconvulsive therapy (ECT), high mobility group box‐1 protein (HMGB1), inflammation, the receptor of advanced glycation end‐products (RAGE)

## Abstract

**Aims:**

High mobility group box‐1 (HMGB1) is one of the damage‐associated molecular patterns produced by stress and induces inflammatory responses mediated by receptors of advanced glycation end‐products (RAGE) on the cell surface. Meanwhile, soluble RAGE (sRAGE) exhibits an anti‐inflammatory effect by capturing HMGB1. Animal models have shown upregulation of HMGB1 and RAGE in the brain or blood, suggesting the involvement of these proteins in depression pathophysiology. However, there have been no reports using blood from depressed patients, nor ones focusing on HMGB1 and sRAGE changes associated with treatment and their relationship to depressive symptoms.

**Methods:**

Serum HMGB1 and sRAGE concentrations were measured by enzyme‐linked immunosorbent assay in a group of patients with severe major depressive disorder (MDD) (11 males and 14 females) who required treatment with electroconvulsive therapy (ECT), and also in a group of 25 age‐ and gender‐matched healthy subjects. HMGB1 and sRAGE concentrations were also measured before and after a course of ECT. Depressive symptoms were assessed using the Hamilton Rating Scale for Depression (HAMD).

**Results:**

There was no significant difference in HMGB1 and sRAGE concentrations in the MDD group compared to healthy subjects. Although ECT significantly improved depressive symptoms, there was no significant change in HMGB1 and sRAGE concentrations before and after treatment. There was also no significant correlation between HMGB1 and sRAGE concentrations and the HAMD total score or subitem scores.

**Conclusion:**

There were no changes in HMGB1 and sRAGE in the peripheral blood of severely depressed patients, and concentrations had no relationship with symptoms or ECT.

## Abstract 2

There were no changes in HMGB1 and sRAGE in the peripheral blood of severely depressed patients, and concentrations had no relationship with symptoms or ECT.

## Graphical abstract

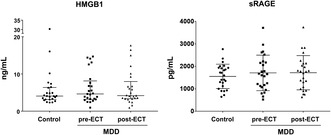

## 1 INTRODUCTION

Depression is a common psychiatric disorder with a lifetime prevalence of 10%–20%, for which it is desirable to develop more effective treatment targets. In recent years, there has been increasing evidence to support an association between depression and inflammatory responses. In particular, it has been reported that inflammatory substances such as inflammatory cytokines and C‐reactive protein (CRP) are elevated in the blood of depressed patients.^1^

Psychological and physical stress induces the release from central and peripheral cells of various inflammatory substances called damage‐associated molecular patterns (DAMPs).^2^ DAMPs bind to pattern recognition receptors (PRRs) and drive activation of NF‐κB signaling, which is thought to cause exacerbation of depressive symptoms by triggering an inflammatory response through the production of inflammatory cytokines.^3^ The receptor of advanced glycation end‐products (RAGE), one such PRR, is a multiligand receptor belonging to the immunoglobulin superfamily. It has two main isoforms: transmembrane RAGE, which is present on the cell surface, and soluble RAGE, which is released into the extracellular space.^4^ It has been clarified that transmembrane RAGE binds to DAMPs and induces an inflammatory reaction, while sRAGE captures DAMPs and exhibits an anti‐inflammatory effect.^5^

High mobility group box‐1 (HMGB1), a representative ligand for RAGE, was originally discovered as a nuclear protein that promotes nucleosome structural maintenance and gene transcription, but it is known to exhibit inflammatory cytokine‐like effects as DAMP.^6^ It has been reported that HMGB1 and RAGE are upregulated in animal models of depression. For example, HMGB1 and RAGE expressions are increased in the brain, including the hippocampal microglia and the cerebral cortex, of chronic unpredictable mild stress and chronic unpredictable stress model mice. It has also been reported that intracerebroventricular administration of HMGB1 induces depression‐like behaviors, such as prolonged immobility time in tail suspension test (TST) or forced swimming test (FST) and decreased sucrose preference.^7^, ^8^, ^9^, ^10^, ^11^, ^12^, ^13^, ^14^ Additionally, the administration of an anti‐HMGB1 antibody was reported to significantly suppress the prolonged immobility time in the FST which was observed in a mouse model of depression induced by neuropathic pain.^15^

Thus, interesting associations about HMGB1 and RAGE have been observed mainly in animal models of depression, but few such studies have been performed using blood samples from human patients with depression. Furthermore, there have been no detailed studies comparing serum HMGB1 and sRAGE concentrations before and after treatment or examining their relationships with depressive symptoms. In this study, we hypothesized that HMGB1 concentrations are increased in depressed patients. Then we measured the serum levels of HMGB1 in depressed patients and also examined whether the levels of its soluble receptor, sRAGE, were altered. We further hypothesized that treatment would restore these balances, and accordingly, we measured HMGB1 and sRAGE concentrations before and after treatment with ECT.

## 2 METHOD

### 2.1 Subjects

Serum samples were collected at the Department of Psychiatry of the National Hospital Organization Kure Medical Center and Chugoku Cancer Center (NHOKMCCCC), Hiroshima, Japan between January 2011 and December 2015. All 25 patients were diagnosed according to the DSM‐IV‐TR. Patients diagnosed as having major depressive disorder (MDD) were recruited from among inpatients who were scheduled for ECT, based on the guidelines of the American Psychiatric Association,^16^ at NHOKMCCCC (*n* = 25, Table 1). Serum samples were collected in the morning (between 7:00 and 8:00 am) before the first ECT session (pre‐ECT) and 2 weeks after the final ECT session (post‐ECT). Twenty‐five subjects with no history of past or current mental disorders were recruited as nondepressed controls, matched for age and gender. Prior to ECT, most MDD patients received antidepressant pharmacotherapy: mianserin (10–30 mg/day; *n* = 3), nortriptyline (100 mg/day; *n* = 4), mirtazapine (15–45 mg/day; *n* = 6), duloxetine (20–60 mg/day; *n* = 5), paroxetine (10–40 mg/day; *n* = 2), imipramine (50 mg/day; *n* = 2), escitalopram (10–20 mg/day; *n* = 5), trazodone (25 mg/day; *n* = 1), or venlafaxine (37.5 mg/day; *n* = 1). The mean imipramine (IMI) dose equivalence according to a previous report^17^ was 153.1 ± 95.9 mg/day. Seven patients received a combination of two antidepressant drugs (imipramine and mirtazapine, *n* = 1; escitalopram and mirtazapine, *n* = 3; duloxetine and mirtazapine, *n* = 3). Angiotensin‐converting enzyme (ACE) inhibitors, angiotensin II receptor blockers, calcium blockers, statins, antidiabetic drugs, chronic renal failure, and diabetes mellitus are factors that may affect blood levels of sRAGE and its ligands; therefore, subjects with these diseases or use of these drugs were excluded.^5^ The protocol for this research project has been approved by a suitably constituted Ethics Committee of NHOKMCCCC (Approval No. 26‐35 and 27‐09) and it conforms to the provisions of the Declaration of Helsinki. Informed consent was obtained from all subjects.

**TABLE 1:** Subjects' demographic data.

	MDD (*N* = 25)	Control (*N* = 25)	*p* Value
Gender (female)	14 (56%)	14 (56%)	1.00^a^
Age (years)	54.2 [48–63]	53.2 [47–61]	0.68^b^
BMI (kg/m^2^)	21.7 ± 3.7	22.8 ± 2.2	0.22^c^
Age of onset (years)	49.4 ± 11.3		
Number of episodes	1.7 ± 2.1		
Number of ECT	10.5 ± 3.2		
HAMD score at pre‐ECT	24.0 ± 7.9		
HAMD score at post‐ECT	5.2 ± 3.0		0.00^d^
IMI equivalence at pre‐ECT (mg/day)	197.5 ± 144.3		
IMI equivalence at post‐ECT (mg/day)	197.5 ± 95.7		1.00^d^

*Note*: Data are shown as the mean ± SD or median with interquartile ranges or number (%).

^a^ Comparison between MDD and control group by chi‐square test.

^b^ Comparison between MDD and control group by Mann–Whitney U‐test.

^c^ Comparison between MDD and control group by *t*‐test.

^d^ Comparison between pre‐ECT and post‐ECT by paired *t*‐test.

### 2.2 ECT procedure

Clinical symptomatic scores were assessed by trained psychiatrists using the 21‐item Hamilton Rating Scale for Depression (HAMD) prior to the first ECT session (pre‐ECT) and after the final ECT session (post‐ECT). ECT was performed according to the procedure of a previous report.^18^ Anesthesia was induced with thiamylal sodium (2–3 mg/kg, i.v.) and suxamethonium chloride (0.5–1 mg/kg, i.v.). The ECT device used was the Thymatron System IV brief pulse square‐wave apparatus (Somatics Inc., Lake Bluff, IL). Electrodes were positioned bilaterally on the frontal‐temporal region. Only one adequate seizure was required for each session, which was defined as an electroencephalographic seizure persisting for more than 25 s with a high amplitude, slow wave, and postictal suppression. The initial stimulus dose was determined using the half‐age method. If an adequate electroencephalographic seizure occurred in one session, the same stimulus energy was used in the next session. When a missed or an inadequate seizure occurred, the patient was restimulated with 1.5–2 times the initial stimulus. The maximum number of stimulations for each treatment session was 2. ECT was administered a maximum of three times per week. If any adverse effects (e.g., cognitive dysfunction or delirium) occurred, the frequency of the ECT schedule was reduced to once or twice per week. ECT continued until the patient was asymptomatic or the attending psychiatrist determined that the patient had obtained the maximum benefit.

### 2.3 Measurement of HMGB1 and sRAGE

The serum HMGB1 level was determined with an HMGB1 ELISA Kit (Shino‐test, JAPAN), the typical detection range is 1–80 ng/mL. The serum sRAGE level was determined with a Quantikine human RAGE immunoassay (R&D Systems, Minneapolis, MN), the typical detection range is 78–5000 pg/mL.

### 2.4 Statistical analysis

Data are shown as mean ± SD or median with interquartile ranges. Normality was tested using the Shapiro–Wilk test (SPSS version 22.0 for Windows, IBM Japan Corporation, Tokyo, Japan). Significant differences between the two groups were evaluated with Student's *t*‐test for normal data or the Mann–Whitney U test for non‐normal data. A chi‐square test was used for categorical variables. Linear regression analysis controlled for age and gender was performed to evaluate the possible relationships between serum HMGB1 and sRAGE levels and the clinical parameters and depressive symptoms.

## 3 RESULTS

### 3.1 Clinical data

The demographic data of the patients and healthy controls are given in Table 1. The sample included 25 each of healthy controls and MDD patients (11 males and 14 females). There were no significant differences in gender, age, or BMI between the control and MDD groups. In the MDD group, there were no significant differences in imipramine equivalents before and after ECT. Total HAMD before ECT indicated moderate to severe depression. Total HAMD after ECT treatment decreased significantly and the mean score was less than seven points in the state of remission.

### 3.2 Serum levels of HMGB1 and sRAGE

**FIGURE 1:**
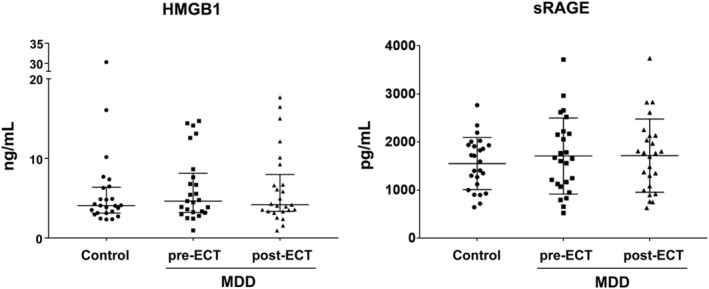
Scatter plot of serum levels of HMGB1 and sRAGE in controls (●), in the MDD group before a course of ECT (pre‐ECT, ■) and in the MDD group after a course of ECT (post‐ECT, ▲). Data are shown as the median with interquartile ranges (HMGB1) or the mean ± SD (sRAGE).

There were no significant differences in HMGB1 and sRAGE concentrations between the control and MDD groups (pre‐ECT; HMGB1, *p* = 0.528; sRAGE, *p* = 0.420) (Figure 1). Linear regression analysis likewise showed no significant difference after adjusting for age and gender (data not shown). In addition, in the MDD group, there were no significant differences in HMGB1 and sRAGE concentrations before and after ECT (HMGB1, *p* = 0.677; sRAGE, *p* = 0.922) (Figure 1). Among the male participants, sRAGE concentrations tended to increase in the MDD group compared to healthy subjects, although the difference did not achieve significance (*p* = 0.074). Finally, linear regression analysis was performed to determine whether there was a correlation between the HMGB1 and sRAGE concentrations after adjusting for age, gender, and BMI, but no significant correlation was found in either the healthy or MDD group (data not shown).

### 3.3 Relationship between serum HMGB1 or sRAGE level and depressive symptoms

The respective correlations of HMGB1 and sRAGE levels with MDD and depressive symptoms (HAMD subitems) were investigated using linear regression analysis, which revealed no correlation between HMGB1 or sRAGE and total score or individual subitems, after adjusting for gender and age (Table 2).

**TABLE 2:** Correlation analysis of HMGB1 and sRAGE levels with HAMD score in MDD.

	HMGB1		sRAGE	
	Partial correlation coefficient	*p*‐Value	Partial correlation coefficient	*p*‐Value
HAMD subitem scores				
Depressed mood	0.392	0.116	0.233	0.372
Feelings of guilt	0.155	0.415	0.264	0.331
Suicide	0.221	0.333	0.285	0.302
Insomnia: early in the night	0.346	0.167	0.007	0.543
Insomnia: middle of the night	0.329	0.189	−0.043	0.537
Insomnia: early hours in the morning	0.395	0.113	−0.254	0.344
Work and activities	−0.169	0.4	0.274	0.317
Retardation	−0.069	0.488	0.008	0.543
Agitation	0.094	0.472	0.256	0.342
Anxiety psychic	0.014	0.507	0.257	0.34
Anxiety somatic	0.136	0.436	0.209	0.403
Somatic symptoms gastro‐intestinal	0.034	0.503	0.166	0.453
General somatic symptoms	−0.232	0.319	0.029	0.541
Genital symptoms	−0.064	0.491	0.012	0.543
Hypochondrials	0.335	0.181	0.167	0.451
Loss of weight	−0.033	0.503	0.111	0.502
Insight	0.229	0.323	−0.116	0.498
Diurnal variation	−0.114	0.456	−0.028	0.541
Depersonalisation or derealisation	−0.027	0.505	−0.086	0.518
Paranoid symptoms	0.36	0.151	0.384	0.169
Compulsive symptoms	−0.015	0.507	0.016	0.543
Total scores of HAMD 21 items	0.222	0.333	0.232	0.374

## 4 DISCUSSION

In the present study, contrary to our hypothesis developed based on prior studies in animal models, there were no significant differences in HMGB1 and sRAGE concentrations in the peripheral blood of severely depressed patients who required ECT. There was no significant change in these concentrations before and after treatment with ECT in the same patients. Additionally, there was no significant correlation between either concentration and the total score or subitem scores of HAMD.

Since injection of recombinant HMGB1 (rHMGB1) into the mouse brain leads to depressive‐like behaviors (prolonged immobility time in TST and decreased sucrose preference), it is possible that HMGB1 may associate with depressive symptoms when the level in the brain is specifically increased.^11^ In animal studies, stress induces both central and peripheral elevations of HMGB1, reflecting systemic inflammation, but the present results indicate no similar phenomenon in humans. One possible reason why similar results were not obtained in this study is due to species differences. In addition, depressed patients exhibit a variety of pathological conditions, whereas animal models of depression are thought to reflect only partial aspects of these pathological conditions.

Previous studies have reported that blood levels of HMGB1 are higher in depressed patients with asthma,^19^ and that patients with high blood levels of HMGB1 during acute cerebral infarction are more likely to experience a depressive state 3 months later.^20^ In addition, the HMGB1‐RAGE pathway is known to be involved in various inflammatory diseases (diabetes, rheumatoid arthritis, inflammatory kidney disease, heart disease, tumor growth, chronic obstructive pulmonary disease).^21^, ^22^, ^23^, ^24^, ^25^ Thus, HMGB1 is involved in depression induced by inflammatory diseases, and it is possible that changes in peripheral blood HMGB1 can be detected in depressive patients with those diseases. Therefore, as in the present study, it is possible that peripheral HMGB1 and sRAGE do not change in depressed patients without concomitant inflammatory diseases.

One report has shown that sRAGE concentrations are significantly lower in depressed patients.^26^ However, distinct from this study, the previous study did not make any assessment before and after treatment or examine the association with symptoms, and it was a univariate analysis. Additionally, the results of the previous study were obtained in Caucasian subjects, whereas the present results were all measured in Japanese subjects and corrected for age and gender. Racial differences in sRAGE expression have been reported previously,^27^ and it is possible that no significant changes would have been observed in Japanese subjects.

It has been reported that the ratio of sRAGE to its representative ligands, such as advanced glycation end‐products (AGE), the AGEs/sRAGE ratio, may be a useful biomarker.^28^ Thus, it would be beneficial to investigate the ratio of sRAGE to its ligands, especially DAMPs, as part of clarifying the relationship between inflammation and depression. Hence, in the present study, we calculated the HMGB1/sRAGE ratio and examined whether this ratio differed between the control and MDD groups and before and after ECT; however, no significant difference was found.

In this study using human peripheral blood, no significant relationship between HMGB1, sRAGE, and depression could be detected. Although only a small number of patients were examined, it should be emphasized that this is the only report that has conducted a detailed examination of before and after treatment comparisons and associations with depressive symptoms in severe depression, excluding the effects of other inflammatory diseases and medicines that affect the expression of sRAGE and its ligands. Future studies should include an increased the number of patients and completely exclude the effects of psychotropic drugs such as antidepressants. It has been reported that sRAGE does not permeate the blood–brain barrier in humans but is locally released into the cerebrospinal fluid (CSF),^29^, ^30^ so it may be possible to find subtle changes in HMGB1 and sRAGE in healthy and depressed patients by using CSF, which is more likely than peripheral blood to reflect conditions in the brain. Although this study focused on HMGB1 and sRAGE, a comprehensive analysis of changes in DAMPs and PRRs, including investigation of the ratio of DAMPs to PRRs, may help elucidate the relationship between depression and inflammation in the future.

## AUTHOR CONTRIBUTIONS

HA, MO‐T, NK, and MT conceived and designed the experiments. WO, KI, and CS collected the samples. HA performed the experiments and analyzed the results. MO‐T, NK, WO, and MT performed the experimental support, analyzed the data, and prepared the figure and tables. MO‐T, NK, SB, TM, and MT revised the paper critically for important intellectual content. HA, MO‐T, NK, and MT wrote the paper.

## FUNDING INFORMATION

This work was supported in part by Policy‐based Medical Service Foundation.

## CONFLICT OF INTEREST STATEMENT

The authors declare no conflict of interest.

## ETHICAL STATEMENT

Approval of the research protocol by an Institutional Reviewer Board: The protocol for this research project has been approved by a suitably constituted Ethics Committee of the National Hospital Organization Kure Medical Center (Approval Nos. 26‐35 and 27‐09).

Informed Consent: All informed consent was obtained from the subjects to participate in the study.

Registry and the Registration No. of the study/trial: N/A.

Animal Studies: N/A.

## Supporting information


Table S1

## Data Availability

The data that supports the findings of this study are available in the Table S1 of this article.
